# New Insights about Enzyme Evolution from Large Scale Studies of Sequence and Structure Relationships*

**DOI:** 10.1074/jbc.R114.569350

**Published:** 2014-09-10

**Authors:** Shoshana D. Brown, Patricia C. Babbitt

**Affiliations:** From the Departments of ‡ Bioengineering and Therapeutic Sciences and; § Pharmaceutical Chemistry, School of Pharmacy, and; the ¶ California Institute for Quantitative Biosciences, University of California, San Francisco, California 94158-2330

**Keywords:** Computational Biology, Enzyme, Molecular Evolution, Multifunctional Enzyme, Protein Evolution, Enzyme Evolution, Enzyme Structure-Function Relationships, Enzyme Superfamily, Networks

## Abstract

Understanding how enzymes have evolved offers clues about their structure-function relationships and mechanisms. Here, we describe evolution of functionally diverse enzyme superfamilies, each representing a large set of sequences that evolved from a common ancestor and that retain conserved features of their structures and active sites. Using several examples, we describe the different structural strategies nature has used to evolve new reaction and substrate specificities in each unique superfamily. The results provide insight about enzyme evolution that is not easily obtained from studies of one or only a few enzymes.

## Introduction

Although we have long assumed that there are many more protein functions in living organisms than fold types (1), *how* a modest number of structural scaffolds (2) have been remodeled by nature to produce the proteins required by living organisms is not well understood. This minireview focuses on functionally diverse enzyme superfamilies, groups of proteins that offer special insight about how nature has solved this challenge.

Functionally (or mechanistically) diverse superfamilies are evolutionarily related sets of enzymes that may be quite diverse in sequence, structure, and overall reaction, but share a conserved constellation of active site residues used for a common partial reaction or chemical capability (3–5). Knowing the fundamental chemical capability and associated substrate substructure(s) that typify each such superfamily constrains the search space for predicting the molecular function of superfamily members of unknown function (unknowns). Comparison among all of the sequences and/or structures in a superfamily can then be used to deduce how evolution has varied these features to produce new enzyme functions from the ancestral structural scaffold. These analyses are valuable for gaining functional clues for the enormous number of sequenced genes that do not have experimental information.

A better understanding of natural enzyme evolution in these types of superfamilies has many other applications as well. For example, understanding how nature has engineered new reactions using the conserved structural features typifying each superfamily could be used to help guide enzyme design in the laboratory (6). Further, assignment of sequences associated with unusual chemical reactions to a superfamily with mechanistically well characterized members may provide clues useful for determining the mechanism of such “outlier” reactions.

Functionally diverse superfamilies represent a significant proportion of the enzyme universe, making up more than one-third of all structurally characterized enzyme superfamilies (7). Because these superfamilies may represent many thousands of sequences and sometimes dozens of different reactions, an inventory of their properties typically requires computational analysis. Many different types of large scale computational studies, focusing on one or multiple superfamilies, have been carried out. See Refs. 8–10 for a few examples. Recently, some of these studies have used network-based approaches (2, 11–13).

**FIGURE 1.**
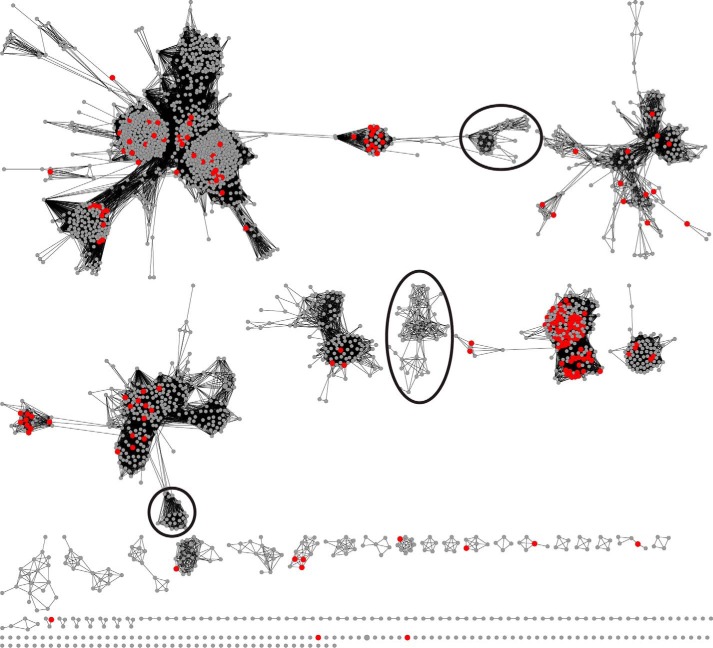
**Representative sequence similarity network for the isoprenoid synthase I superfamily that is available from the Structure-Function Linkage Database (SFLD) (62).** Each node (*circle*) represents a group of 1–732 sequences, where each sequence in a node is at least 50% identical to a seed sequence that defines that node (computed using the CD-HIT program (63)). The 2,499 nodes in this network represent over 16,000 sequences. Each edge (*line*) between two nodes indicates that the sequences represented by the connected nodes have a BLAST similarity score with an average −log(E-value) of 30 or more significance. At this −log (E-value) cutoff, alignments have an average length of 273 amino acids, and an average percent identity of 31%. Nodes are laid out in Cytoscape using the yFiles organic layout. A node is colored *red* if at least one constituent sequence represented by that node has a functional annotation in the Swiss-Prot database. A node is colored *gray* if no sequence in that representative node has a functional annotation in Swiss-Prot. Several clusters of nodes where no corresponding sequence has a functional annotation in Swiss-Prot are indicated with *black ovals*.

Reflecting this relatively new approach, sequence similarity networks are used in some figures in this review (see Figs. 1 and 4) to enable exploration of structure-function relationships in enzyme superfamilies from a large scale perspective. In these networks, nodes represent one or more proteins, and edges between them represent a measure of sequence or structural similarity. Although not a substitute for phylogenetic trees, similarity networks provide several advantages over trees and multiple alignments for developing new hypotheses about the evolution of functional features in superfamilies. They are quick to construct, do not require an accurate multiple sequence alignment, and can summarize in one network relationships among thousands of sequences. The networks can also be visualized and interactively manipulated and explored using such software packages as Cytoscape (14). Although they are not based on an explicit evolutionary model, initial validation studies show that similarity networks correlate well with results from phylogenetic trees (15).

We illustrate here some major themes emerging from large scale studies of functionally diverse enzyme superfamilies that impact our understanding of the evolution of enzyme function. First, studies of a number of these enzyme superfamilies suggest that experimental knowledge of their functions is sparse and that we know very little about the functions of a large proportion of enzymes in each. This lack of knowledge limits our understanding of the evolution of new reactions in significant ways. Second, the patterns of structural variation associated with the evolution of diverse functions in these superfamilies are many and varied and include, for example, structural reorganization of domains, addition of inserts, and even major modifications in active site architecture. Many of these patterns are difficult to deduce from small scale comparisons. Third, deducing how differences in reaction and substrate specificity have evolved within a functionally diverse superfamily can be complicated by issues that are challenging to address. Functional promiscuity (2) and evolutionary invention of the same reaction more than once from intermediate ancestors in a superfamily phylogeny (16–18) provide relevant examples.

## We Know Very Little about Structure-Function Relationships in Large Enzyme Superfamilies

When examining the available functional information for a superfamily, one of the most striking observations is how much we do not know. This is due in part to the rapid increase in sequence information that continues to accrue at a rapid rate. As a result, the members of many superfamilies now contain many thousands of sequences for which no functional information is available. Even in well studied superfamilies, there are large swaths of protein space where reliable predictions of even general functional features may be difficult. For example, the sequence similarity network in Fig. 1 shows members of the “isoprenoid synthase I” superfamily (19, 20) mapped with functional annotations from the Swiss-Prot database (21). Swiss-Prot annotations are reviewed by curators, preferably based on experimental information, and have been found to be highly accurate when compared with annotations from other major protein databases (22). Although many of the sequences in Fig. 1 have a functional annotation in Swiss-Prot (*red nodes*), there are many other nodes that do not, including sequences in the large clusters highlighted in the figure. With so many of these sequences representing unknowns, deducing their contributions to our understanding of the evolution of function in this superfamily will remain challenging, perhaps for years to come. This trend is general, with 20% of protein domains in the Pfam database (23) annotated as “domains of unknown function”. A recent study of model bacterial organisms suggests that many of these domains of unknown function are essential proteins (24).

In other superfamilies, even those of broad importance to many organisms, the proportion of unknowns can be even higher. In the cytosolic glutathione transferases, the large majority of sequences are unknowns, with only a minority of superfamily members confirmed to catalyze glutathione transferase-like reactions (16). Knowledge of the physiological reaction(s) catalyzed by these important and heavily studied enzymes is even more sparse as most enzymes experimentally confirmed to catalyze glutathione transferase activity have relied on assays using synthetic compounds as substrates. Likewise, only a relatively few of the enzymes of the “radical *S*-adenosyl methionine (SAM)” superfamily have been structurally characterized (25, 26). As many of the 50,000 nonredundant sequences of this superfamily are highly divergent from each other, understanding their evolution from the currently available structural information is still substantially limited. Further complicating this task, the few structures that are available exhibit such major variations that inference of general features of even the folds of many unknowns is difficult.

Another confounding issue for understanding the evolution of enzyme superfamilies is that the proteins of known function are often not evenly distributed across the sequence space of a superfamily. This is in part due to bias in targets chosen for experimental characterization, with characterization favored for proteins from model organisms or from organisms such as pathogens where the need for functional knowledge may be especially compelling. In the cytosolic glutathione transferases, for example, the human enzymes are far better studied than those from other species. Moreover, high throughput experiments, in which a very small number of studies currently account for a significant proportion of electronically available annotations, show biases in the types of functional information they provide as well as in the types of proteins that are targeted (27).

## Many Types of Structural Variation Accompany Evolution of New Functions

How common functional features are retained during evolution while also allowing for the sequence and structural variation required to produce new reactions remains a major question for understanding enzyme evolution in functionally diverse superfamilies. Variations in oligomeric state and protein-protein interactions among sets of homologous proteins are of course well known. Large scale studies of enzyme superfamilies have more recently begun to reveal in greater detail broad patterns by which the divergence in function of each unique superfamily may be accompanied by significant structural modifications. Here, three different superfamilies illustrate strategies nature has used to maintain the fundamental chemistry “hard-wired” into a structural and active-site architecture while enabling the evolution of many different reactions.

**FIGURE 2.**
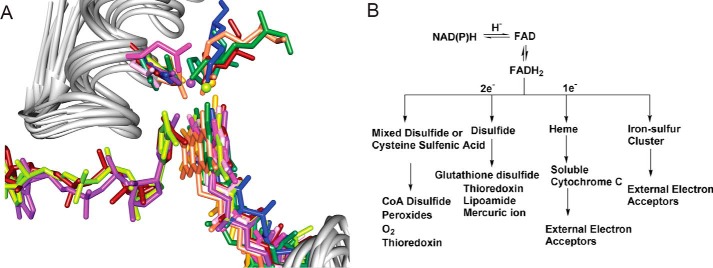
**Transfer of electrons from members of the tDBDF superfamily to acceptors.** *A*, superimposed active sites showing 10 members of the tDBDF superfamily. The cofactors and conserved side chains important for stabilizing the isoalloxazine and nicotinamide ring complex are shown in color with each color representing a different reaction family structure. Water residues involved in stabilizing the complex are shown as *balls. B*, superfamily members can transfer electrons to acceptors one or two at a time. Intermediate acceptors can be small molecules or proteins, which in turn transfer electrons to a variety of small molecule acceptors or external protein partners. Figure and legend adapted from Ref. 29.

The cofactor-dependent “two dinucleotide-binding domains flavoproteins” (tDBDF)^2^ superfamily is composed of many different reaction families that include several types of monooxygenases, reductases, and dehydrogenases. Comparison of their sequences and structures illustrates how variations in protein-protein interactions can enable a diverse set of overall reactions while the specific organization of the cofactors within the active site is stringently constrained by an active site architecture required for binding the dinucleotide cofactors (28–30). This ensures that all enzymes of the superfamily share a unidirectional electron flow from the *re*-side to the *si*-side of the isoalloxazine ring of the FAD cofactor so that electron acceptors unique to each member family access the FAD cofactor from the *si*-side of the isoalloxazine ring (Fig. 2*A*). Diversity in the functions of the different reaction families has evolved in part by pairing the delivery of electrons out of the tDBDF member active sites with varied electron acceptors presented via protein-small molecule or protein-protein interactions (Fig. 2*B*) (29). Many of the penultimate or ultimate acceptor proteins come from different fold classes, resulting in a number of solutions for the evolution of these important oxidation/reduction systems.

The “vicinal oxygen chelate fold” (VOC) superfamily represents a quite different structural paradigm (31). All of these enzymes share a common βαβββ fold module that provides the environment for metal coordination promoting direct electrophilic participation of the metal ion in catalysis (with the notable exception of the non-enzymatic bleomycin- and mitomycin-binding proteins (32)). This common module is combined and permuted in at least six distinct ways in the different enzymes in the superfamily (33), each associated with different types of reactions, including nucleophilic opening of epoxide, oxidative cleavage of a C–C bond, isomerization, and epimerization.

Another type of structural variation in enzyme superfamilies involves addition of inserts of varying size and functional roles within a conserved core domain. These can play a functional role in enabling diversity in overall reactions while maintaining the chemical capability common to all members of the superfamily. In the “haloalkanoic acid dehalogenase” (HAD) superfamily, an aspartate nucleophile, which forms a covalent intermediate with the substrate, is well conserved (34). Other catalytic residues are found in somewhat different configurations depending on the function of the enzyme, but are also relatively well conserved. Although the core Rossmann fold provides much of the fundamental chemistry that typifies the superfamily, many substrate-binding residues are contributed by variable capping domains, with the active sites of these enzymes situated between the core and capping domains. The cap domains may be inserted at two different points within the conserved core structure and can come from different fold classes (34–36). Although the capping domains clearly play a role in function, functional type does not cleanly correlate with cap type. Thus, although co-evolution between core and cap domains offers hints about how this large superfamily has evolved structural diversity, mapping of these variations to functional properties remains a difficult challenge.

**FIGURE 3.**
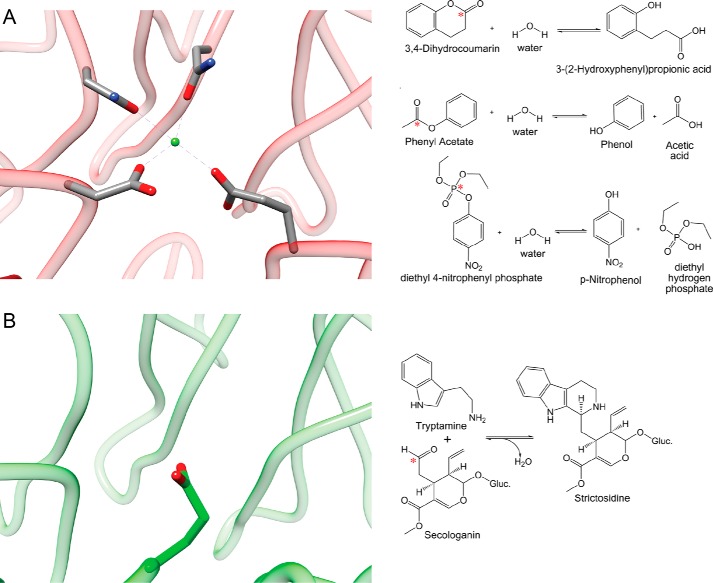
**A related catalytic strategy unites the strictosidine synthase enzymes with the rest of the N6P SF.** *A*, *left panel*, active site of diisopropylfluorophosphatase (Protein Data Bank (PDB) id: 2gvv). The four metal-binding ligands conserved in the majority of superfamily members are shown coordinated to a divalent metal ion. *Right panel*, examples of reactions catalyzed by characterized metal-dependent proteins. B, *left panel*, active site of strictosidine synthase (PDB id: 2fpb). *Right panel*, the metal-independent strictosidine synthase reaction. (Figure and legend adapted from Ref. 37 with permission.) In both *right panels*, a *red asterisk* indicates the electrophilic atom that is attacked in the reactions catalyzed by characterized members of the superfamily.

Profound variations in the active sites of members of functionally diverse superfamilies may also allow for diversity in the overall reactions catalyzed across a superfamily. The members of the “nucleophilic attack, 6-bladed β-propeller” (N6P) superfamily share a general catalytic strategy involving nucleophilic attack on an *sp*^2^-hybridized electrophilic atom (37). One major sequence similarity subgroup of the superfamily includes only a few characterized proteins that all catalyze esterase, lactonase, and/or phosphotriesterase reactions; a second subgroup is predicted to catalyze arylesterase-like reactions, typified by only one well characterized organophosphatase reaction, human paraoxonase (38). In the experimentally and structurally characterized proteins of these two subgroups, four conserved active site residues serve as ligands to a divalent metal ion required for catalysis in these hydrolytic reactions (39–42) (Fig. 3*A*). These conserved metal ligands can be identified from sequence comparisons so that the structural and active site similarities predicted for the sequences in these two subgroups suggest that most of the unknowns likely catalyze similar types of hydrolytic reactions.

A third sequence-similar subgroup of ∼600 sequences, the “strictosidine synthase-like” proteins, was named for the function of the only experimentally characterized sequences in the subgroup. These enzymes catalyze the metal-independent condensation of tryptamine and secologanin to form strictosidine (43) (Fig. 3*B*). Because the sequences of this subgroup are more similar to characterized strictosidine synthases than to the sequences of the other two subgroups, they have been annotated in public databases as strictosidine synthases or strictosidine synthase-like proteins. It was not until these sequences were examined as part of a large scale analysis of the entire superfamily (37) that it became clear that the experimentally characterized strictosidine synthases were outliers even in the so-called strictosidine synthase-like subgroup. Unlike the seven proteins experimentally confirmed to catalyze the strictosidine synthase reaction, the huge majority of the other sequences in this subgroup appear to conserve four metal-binding ligands, and are thus more likely to catalyze hydrolytic reactions rather than the condensation reaction catalyzed by strictosidine synthase (Fig. 3*B*). Indeed, a strictosidine synthase-like protein conserving only three of the four typical metal-binding ligands and identified from phylogenetic analysis to be among the most similar to the experimentally characterized strictosidine synthases was shown to have hydrolytic activity, but no detectable strictosidine synthase activity (37).

The evolutionary trajectory resulting in both the contemporary hydrolytic enzymes and their metal-independent strictosidine synthase homologs remains a mystery. Although both catalytic types can be assigned to the same superfamily based on sequence and structural similarities, the substantive differences in their active sites offer stunning evidence for how little we understand about how new enzymatic reactions evolve. The most parsimonious explanation for the results of this study suggests that strictosidine synthase may have evolved from a metal-dependent ancestor catalyzing hydrolytic chemistry. However, a later comparison of proteins of the larger Pfam clan to which the N6P superfamily belongs suggests that most of those enzymes *lack* the four metal-binding residues that might be expected of a metal-dependent common ancestor (44), raising questions about this simple hypothesis and suggesting a more complicated path for the evolution of these enzymes.

## Challenges for Understanding the Evolution of Varied Functions in Functionally Diverse Superfamilies

Because the different reaction families of a functionally diverse enzyme superfamily all “look alike” with respect to superfamily common active site features, they are difficult to annotate and easy to misannotate (22). Understanding how their different functions evolve while conserving a fundamental chemical strategy is yet more difficult, in part because each superfamily is unique with respect to the linked sequence, structural, and functional features its members share and the ways in which those sequences and structures have been modified by evolution for new functions.

Two other important themes complicate our understanding and at the same time offer new clues about the ways that new reactions may evolve. The first is functional promiscuity, which offers insight about the capabilities of the same active site to support different reactions. The second comes from observations that the same reaction can evolve independently from different intermediate ancestors in a superfamily tree.

### Promiscuity and “Moonlighting” Enzymes

Catalytic promiscuity, the ability of an enzyme to catalyze different types of reactions using the same active site, has been observed in many enzymes. The seminal study by O'Brien and Herschlag (45) described this phenomenon in multiple systems and tied it to the evolution of new activities via enzyme duplication, a concept further elaborated by others, for example, in Ref. 46. Although some promiscuous enzymes evolved to catalyze the same reaction with different substrates, such as cytochrome P450s, others catalyze reactions that appear to be quite different from each other. The *o*-succinylbenzoate synthase (OSBS) enzyme from *Amycolatopsis* sp., a member of the “enolase” superfamily, was originally characterized as an *N*-acylamino acid racemase (47). This annotation was propagated to other related sequences, and only later was it determined that the biologically relevant function of the original enzyme was actually OSBS (48). Other enzymes from this superfamily have now been characterized that catalyze both the OSBS and the *N*-succinyl amino acid racemase reactions, and the evolution of both reactions continues to be a topic of investigation (49–51).

The concept of moonlighting enzymes, describing additional structural or regulatory functions performed in addition to their catalytic functions, has also provided novel insight about the evolution of new reactions from existing structures. An important early example describes the conscription of several different enzymes to serve roles as eye lens proteins (52). Enzymes from functionally diverse superfamilies have also been shown to play moonlighting roles. For example, the glycolytic enzyme enolase, another reaction family of the enolase superfamily, plays many moonlighting roles relevant to human health and disease (see Refs. 53 and 54 for some examples). Broader inventories of moonlighting proteins have also recently been collected (see Refs. 55 and 56 and references therein).

As observations of both promiscuous and moonlighting enzymes continue to increase, it seems likely that both phenomena may be far more widespread than previously thought, although many additional studies will be needed to obtain more accurate estimates of their prevalence. The widespread incidence of promiscuous or multifunctional enzymes also suggests that definitions of functional boundaries may be difficult to determine, even with the aid of similarity clustering of large numbers of sequences (see, for example, Refs. 13 and 57). As a corollary, sorting out the evolutionary path by which these functional types emerged may not accurately predict functional boundaries either, especially in functionally diverse superfamilies. Indeed, the idea that each enzyme has a single, specific function may be a more artificial construct than has been recognized. Alternatively, enzyme function space may perhaps be better described as a continuum rather than a set of discrete definitions.

### Invention of the Same Reaction within a Superfamily Phylogeny

**FIGURE 4.**
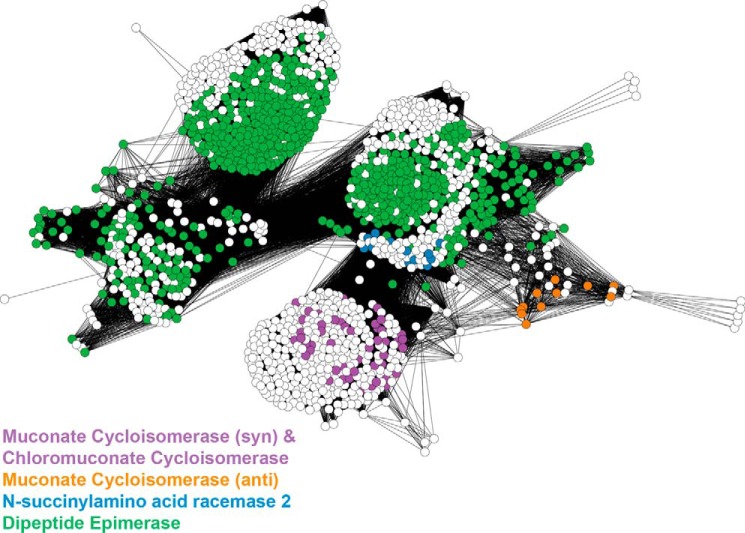
**Full sequence similarity network for a subset of the enolase superfamily, including the two muconate cycloisomerase families and their closest neighbors.** Each node (*circle*) represents a single sequence, and edges (*lines*) represent BLAST connections with a −log (E-value) of 47 or more significant. Nodes are laid out using the yFiles organic layout and colored by SFLD family assignment.

Convergent evolution, where unrelated enzymes have evolved to catalyze the same overall reaction, has been well studied. (For a recent review, see Ref. 58 and references therein.) Perhaps less widely appreciated is the propensity for proteins with the same, or similar, functions to be invented multiple times from different starting points within a superfamily (13, 16–18, 59–61). Fig. 4 shows a sequence similarity network of a subgroup of enzymes of the enolase superfamily, including the two different families that catalyze the cycloisomerization of muconate. These two families are distinct from each other in the phylogenetic tree of the subgroup to which both belong (49), suggesting that they evolved from different progenitors within the subgroup. Supporting this suggestion, experimental work shows that the stereochemical course of the reaction differs between the two families due to different modes of substrate binding, which result in opposite faces of the enolate anion intermediate being presented to the conserved Lys acid catalyst (17). In the same subgroup, two different families of enzymes that catalyze an *N*-succinyl amino acid racemase reaction have also been identified (18).

Because all enzymes in each functionally diverse superfamily already have the chemical machinery required for a critical part of the reaction, it may not be surprising that enzymes catalyzing the same overall reaction might evolve from different precursors within the superfamily. This has important implications both for understanding enzyme evolution and for predicting the functions for proteins in functionally diverse superfamilies. Knowing that a particular function is found in one region of a phylogenetic tree does not preclude the same function from occurring in another, quite distant, area of the tree. Further, knowing that two enzymes catalyze the same overall reaction does not necessarily mean that they are close homologs, even if they belong to the same superfamily.

## Conclusions and Future Directions

In the breadth of their organismal representation, the range of biochemical reactions they catalyze, and the many ways nature has reused each ancestral scaffold for many different functions, functionally diverse enzyme superfamilies offer a powerful model for understanding how the enzymes required for life have evolved. By determining the conserved structure-function paradigm represented by each of these “privileged scaffolds,” we can begin to sort out the structural variations nature has used to evolve a wide array of different chemical reactions and tailor each of them for their specialized biological roles.

Analysis of such superfamilies is daunting, however, as each may contain tens of thousands of sequences, most of which are of unknown reaction or substrate specificities. Thus, the challenges even for managing the data, let alone inferring the functional repertoire each superfamily supports, may appear at first glance to be insurmountable. Offsetting the obvious impossibility of experimentally characterizing the molecular functions of the still rapidly growing volume of newly discovered enzyme sequences, we propose that the context provided by large scale computational characterization of these superfamilies will lead to new types of hypotheses about their structure-function relationships that cannot be accessed by comparison of only a few homologous enzymes. Even in the early forms in which this technology has been used, protein similarity networks have already been shown to provide structure-function mapping on the scale required. Future work by both the biochemical and the computational communities will improve both the robustness and the interpretability of this approach and expand its applications to address pressing issues that range from choosing experimental targets for answering many types of questions to exploiting our understanding of natural evolution to aid in engineering new reactions in the laboratory.

## Footnotes

2 The abbreviations used are:

tDBDF: two dinucleotide-binding domains flavoproteins
N6P: nucleophilic attack, 6-bladed β-propeller
OSBS: *o*-succinylbenzoate synthase.
